# Structure-Property Relationship for Different Mesoporous Silica Nanoparticles and its Drug Delivery Applications: A Review

**DOI:** 10.3389/fchem.2022.823785

**Published:** 2022-03-14

**Authors:** Parya Kazemzadeh, Khalil Sayadi, Ali Toolabi, Jalil Sayadi, Malihe Zeraati, Narendra Pal Singh Chauhan, Ghasem Sargazi

**Affiliations:** ^1^ Department of Chemistry, Lorestan University, Khorramabad, Iran; ^2^ Department of Chemistry, Young Researchers Society, Shahid Bahonar University of Kerman, Kerman, Iran; ^3^ Department of Environmental Health Engineering, School of Public Health, Bam University of Medical Sciences, Bam, Iran; ^4^ Department Environmental Engineering, University of Zabol, Zabol, Iran; ^5^ Department of Materials Engineering, Shahid Bahonar University of Kerman, Kerman, Iran; ^6^ Department of Chemistry, Faculty of Science, Bhupal Nobles’ University, Udaipur, India; ^**7**^ Noncommunicable Diseases Research Center, Bam University of Medical Sciences, Bam, Iran

**Keywords:** mesoporous, silica, nanoparticles, properties, drug delivery

## Abstract

Mesoporous silica nanoparticles (MSNs) are widely used as a promising candidate for drug delivery applications due to silica’s favorable biocompatibility, thermal stability, and chemical properties. Silica’s unique mesoporous structure allows for effective drug loading and controlled release at the target site. In this review, we have discussed various methods of MSNs’ mechanism, properties, and its drug delivery applications. As a result, we came to the conclusion that more *in vivo* biocompatibility studies, toxicity studies, bio-distribution studies and clinical research are essential for MSN advancement.

## Introduction

One of the most important purposes pursued by nanotechnology is the production of nanoparticles that have the desired distribution of drug in the body (El-Boubbou, 2018; Tandel et al., 2018). Over the past two decades, researchers have concluded that a vital step in the development of drugs is to focus on designing new drug delivery systems (NDDS) (Martínez-Ortega et al., 2019). Ideally, all new systems should improve the stability, absorption, drug concentration, and long-term release of the drug in the target tissue. In addition (Djekic et al., 2019), reducing the number of drug injections to increase patient comfort (Reibaldi et al., 2019), advanced drug delivery systems (Bhatia, 2016), pharmacokinetics of proteins (Turner and Balu-Iyer, 2018), and peptides (Hughes, 2005) that are usually low in half-time should be considered. The ultimate goal of the drug research is to safely transfer the drug to the suitable location in the body at the right time (Jahangirian et al., 2017; Yin and Zhang, 2020). However, for many drugs, these ideals are often impossible. For example, the oral method is usually the best way to use a drug due to non-invasiveness, but peptides and proteins will be absorbed and effectiveness will be reduced due to the acidic properties of the stomach, as well as the effects of the first transfer of the liver, such as drug loss due to metabolic processes prior to systemic rotation and resistance by the intestine (Ahmed and Aljaeid, 2016; Khdair et al., 2016). Finally, its accessibility will be greatly diminished. Nanotechnology, with the elimination of many problems with traditional drugs, allows the application of oral drugs that were previously not usable. In some cases, co-administration of the drug with nanoparticles can increase the bioavailability of the drug in a way that is useful for oral use (Chen et al., 2018). Nanoparticles protect the drugs that are susceptible to degradation in the body and give more durability to the drug’s presence in the blood, attach the drug to the target tissue, release the drug in the target site, and increase the efficacy of the drug several times over (McClements, 2018; Paunovska et al., 2018). The main roles of the preparation of nano-drug delivery systems are to control particle size (Cooley et al., 2018), surface properties (Banerjee et al., 2016) and release of the drug in a good therapeutic dose (Kamaly et al., 2016). Generally, by fabricating silica, the micelles self-assemble around a template and then remove the template using a suitable method such as calcination The design and manufacture of controlled drug release systems can be very helpful in cancer drug therapies. So far, many substances have been introduced as drug release systems, among which biodegradable polymer materials (Goetjen et al., 2020), ceramic materials such as hydroxyapatite (Martínez-Vázquez et al., 2015), and calcium phosphates (Kapoor et al., 2015) can be mentioned. Recently, mesoporous materials have attracted the most attention in this regard. In fact, the porosity of silica mesoporous materials allows biologically active molecules of different sizes to locate in the cavities of these materials (Vallet‐Regí et al., 2007). Also, the regular porosity of these materials makes it possible to achieve a convenient loading and release rate (Anglin et al., 2008). Conversely, as the adsorption of molecules into mesoporous (Manzano and Vallet-Regí, 2019) is a surface phenomenon and the specific surface of these materials also results in the absorption of more active biological molecules (Manzano and Vallet-Regí, 2019). What is important in designing a drug release system is biocompatibility and biodegradability (Jindal et al., 2019). Recent research is based on the development of drug delivery systems that are stable in structure and capable of carrying large volumes of drugs without the problem of early release to target tissues or even small intracellular organs (Manzano and Vallet-Regí, 2019). Among many of the materials that have been investigated in terms of their stable structure for drug delivery, silica mesoporous nanomaterials are known to be biocompatible with defined structures and certain surface specific properties (Dudarko et al., 2019). Silica mesoporous is known as a selective material for the biological applications of inorganic nanoparticles. Typically, silica meso-nanostructure coated with semiconductor quantum dots, such as high-stability cadmium sulfide and selenide (Sharma et al., 2019; Tarrahi et al., 2019), has the potential for chemical change and high biocompatibility that can be used for many diagnostic biomedical applications. In addition, meso silica nanoparticle can be applied to increase the biocompatibility of several drug delivery systems, such as magnetic nanoparticles (Vallet-Regí et al., 2018), biopolymers (Nairi et al., 2018) and micelles (Zhang et al., 2019). In this article, we have reviewed and studied the introduction of various types of silica mesostructure and their application in various drug delivery processes, the advantages of the application of silica nanoparticles in drug delivery systems, and biocompatibility and mechanism reception by the host cell.

## Mesoporous Silica Materials and its Synthetic Method

**FIGURE 1:**
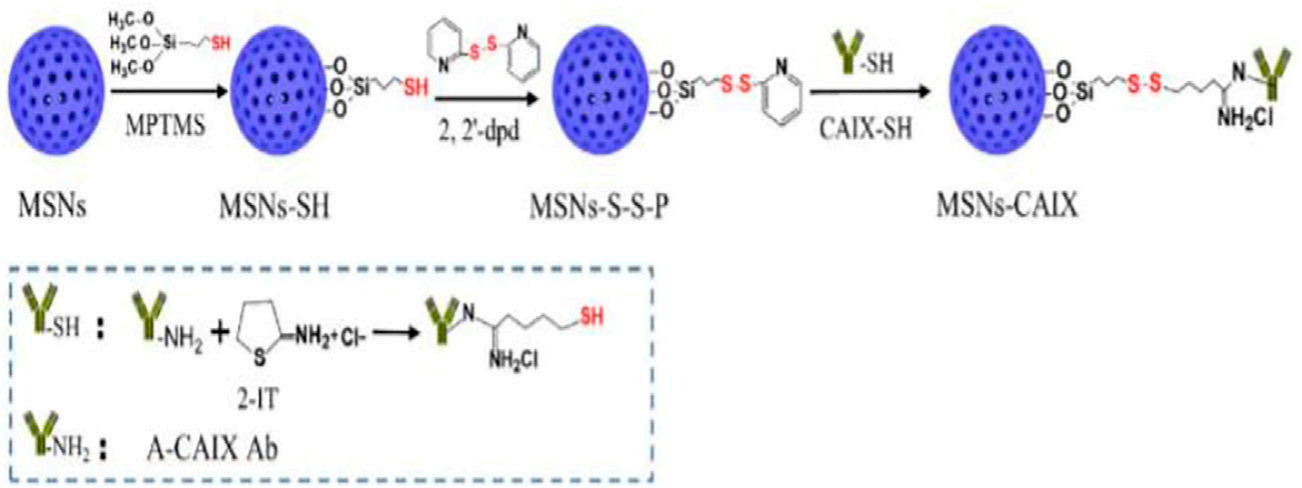
Preparation route for CAIX guided mesoporous silica nanoparticles (Chen et al., 2020).

In general, the porous nanoparticles are divided into three groups according to their size: Microporous (pore size: <2 nm), mesoporous (pore size: 2–50 nm) and macroporous (pore size: >50 nm) (Vallet-Regí, 2012). The mesoporus silica was produced in two steps: first, the micelles self-assembled around a template and then the template was removed *via* calcination (Figure 1). Mesoporous nanomaterials with a well-defined architecture have a high density of silanol (Si-OH) functional groups at their surface that can be modified with a wide range of organic groups to stabilize biomolecules and other applications (Wang, 2009).

Chen and coworkers developed the “DOX@MSNs-CAIX” targeted and redox-responsive drug delivery system, in which MSNs were used as the vehicle for loading chemotherapeutic drug doxorubicin (DOX) and CAIX grafted on MSNs by disulfide bonds. MSNs-CAIX are a promising medication delivery method for cancer treatment with a specific target (Meynen et al., 2009).

**FIGURE 2:**
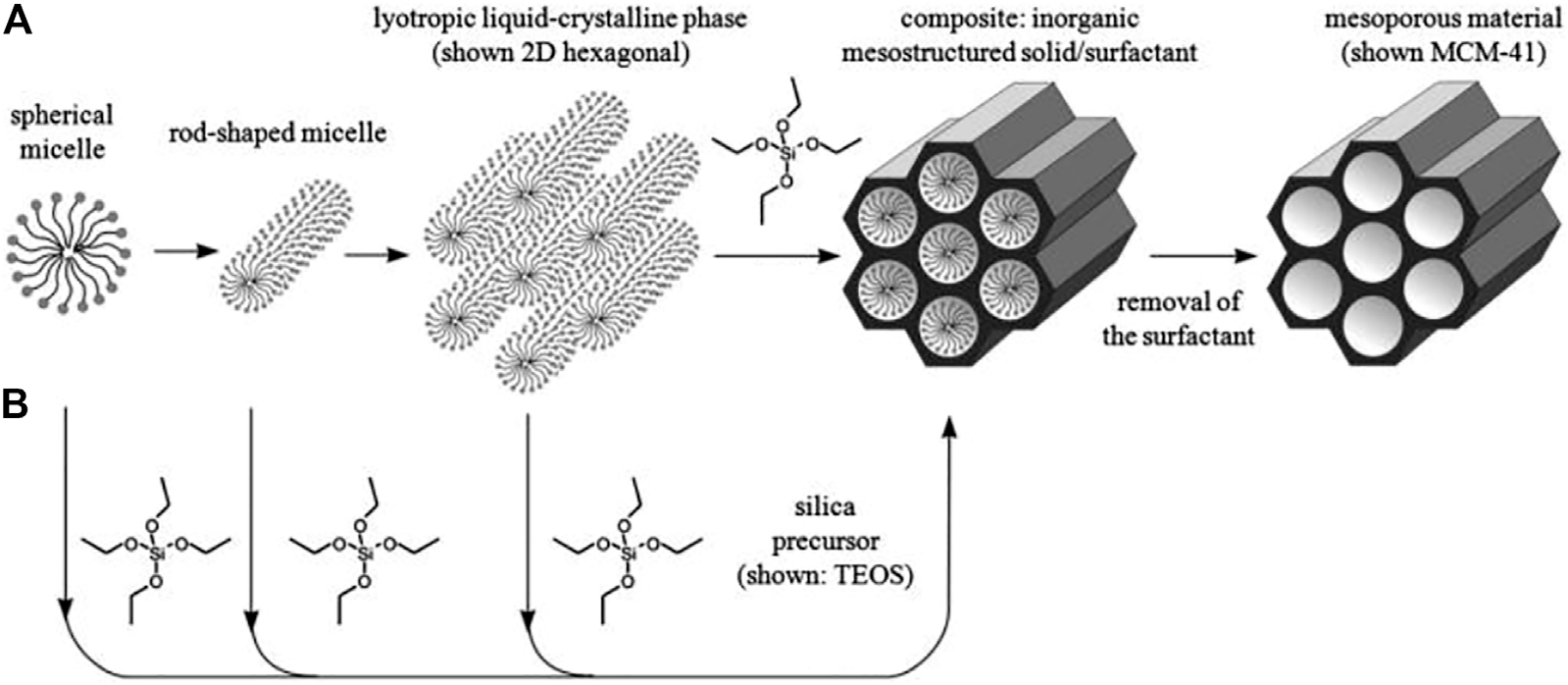
Possible pathways for the synthesis of mesoporous materials **(A)** Liquid crystal templating and **(B)** self-assembly.

In some cases, additional functional groups penetrate into the mesoporous nanoparticles due to fills the silica walls, and consequently reducing the size of the pore and drug loading, despite the fact that this controls drug release. Modification of the nanoparticles surface with a variety of functional groups can cause changes in electrostatic forces, hydrophilic or hydrophobic forces, and internal reactions of the drug and the matrix (Meynen et al., 2009). Various methods for producing silica mesoporous nanomaterials have been reported that show, with sufficient knowledge of preparation methods, pore size engineering, morphological control, and structural properties, that these materials can be of good quality. In addition, the manufacturing of these materials by modifying different of agents, such as a diversity of surfactants, acquires the specific mechanism and the internal reaction of silica with template molecules (Wang et al., 2016; Yi, 2021). For example, through direct reaction of S^+^I^−^, they occur between a positively charged molecular based organized system (MOS) activating surface ion by and a negatively charged silicate source. Two kinds of mechanisms including liquid crystal templating, self-assembly mechanisms have been proposed for mesoporous synthesis (Meynen et al., 2009). Figure 2 presents an overview of possible pathways for their synthesis.

These materials can also be manufactured using surface activators called polymeric based organized systems (POS) through the indirect reaction of template with a silicate source in an acidic media. Conversely, the interaction between the MOS and the inorganic silicate source (S^0^I^0^) leads to the formation of hollow mesoporous silica (HMS) materials (Meynen et al., 2009). Many factors such as pH, salts, inflammasome, co-solvents, co-surfactants, concentration, specific silicate source, solvent, and temperature are involved in the organization of mesopores (Zhao et al., 2000). In a typical method of preparation of mesosilica materials, the dissolution of template molecules in the solvent and the addition of a silicate source such as tetraethoxy ortho silane (TEOS), meta silicate (Na_2_SiO_3_), and gaseous silica are performed. After some time at a certain temperature, the hydrolysis and condensation processes begin. Finally, the resulting product is washed and dried, and by removing the mold using calcining, the silica mesoporous is obtained (Kim et al., 2000).

Mesoporus silica nanoparticles have shown to have excellent properties for biomedical applications. These properties includes porous ordered and well aligned structure, which shows fine control of drug delivery and release kinetics; larger surface area and pore volume, which shows high potential for molecule loading and dissolution enhancement; tunable particle size in between 50 and 300 nm which is suitable for facile endocytosis by living cells; and silanol-contained surfaces have two functional surfaces with cylindrical pore surface, exhibit better control over drug loading and release; and have excellent biocompatibility.

The oral route is the most popular route for drug delivery, despite the fact that many medicines, particularly highly pH- and/or enzymatic biodegradable peptide substances, are extremely difficult to manufacture and obtain efficient intestine absorption. Only if the drug 1) is substantially present as a solution in the gastrointestinal tract, 2) is able to penetrate through the intestinal mucus, 3) overcomes the various gastrointestinal barriers, and 4) provides an effective therapeutic dose that is efficient for systematic absorption of an active substance, is oral ingestion possible. As a result, improving the oral bioavailability of poorly soluble medications is still a major challenge for the pharmaceutical industry. Despite the fact that several traditional drug carriers have solved some of the problems associated with the oral delivery of poorly soluble medicines, only a few have fulfilled commercialization requirements. Due to these limitations, scientists have begun to rethink their methods for targeted drug delivery systems and researchers have begun looking for alternate vectorized carriers (Florek et al., 2017).

Lee et al. synthesized MSNs with varying concentrations of positive surface charges (Lee et al., 2008). The positive surface charge was achieved by directly co-condensing a TA-silane and tetraethoxysilane (TEOS) in the presence of a base as a catalyst and inserting trimethylammonium (TA) functional groups into the framework of MSN (MSN–TA). These MSN–TA samples have well-defined hexagonal structures with an average particle diameter of 100 nm, pore size of 2.7 nm, and surface area of about 1,000 m^2^ g^−1^. Anionic drug molecules, Orange II (a fluorescent tracing molecule), and sulfasalazine (an anti-inflammatory prodrug used for bowel disorders) were successfully loaded into these MSN–TA samples and persisted within the MSN–TA in an acidic environment (pH 2–5) (Li et al., 20211196). Guha and others developed poly (methacrylic acid-co-vinyl triethoxylsilane) coated MSN with better hypoglycemic effect for insulin delivery (Guha et al., 2016). Ang and coworkers have reported the synthesis of MCM-41 nanoparticles using sol-gel method for oral delivery of MCC7433 and pretomanid and its surface was modified with phosphonate and amino groups (Ang et al., 2021). The impact of various structural features of MSNs on protein loading, protection, and delivery performance have been reviewed by Xu and coworkers. They also discussed the current state of MSN research in enzyme mobilization, catalysis, intracellular delivery, extracellular delivery, and antimicrobial protein delivery (Xu et al., 2019). Janjua and coworkers have developed a room temperature procedure for synthesizing ultra-small silica nanoparticles with large pore sizes that can load high amounts of chemotherapeutic medicines and attach a targeting moiety to their surface for the first time. To accomplish additional active targeting, the nanoparticles were coupled with lactoferrin (>80 kDa), whose receptors are overexpressed by both the blood-brain barrier and glioblastoma (Janjua et al., 2021).

Meka et al. studied the solubility, permeability, and anti-cancer activity of vorinostat encapsulated within MSNs with various functional groups. When vorinostat was encapsulated in pristine MSNs, its solubility was increased by 2.6 fold when compared to the free drug (MCM-41-VOR). When MSNs were treated with silanes with amino (3.9 fold) or phosphonate (4.3 fold) terminal functional groups and solubility were increased even further. Also, MSN-based formulations considerably improved vorinostat permeability into Caco-2 human colon cancer cells, particularly MSNs modified with an amino functional group (MCM-41-NH_2_-VOR), where it increased by fourfold (Meka et al., 2018).

## Types of Silica Mesoporous Systems

The meso-silica, due to their high thermality, chemical stability, high surface area, and good compatibility with other materials meso-silica systems, they have found wide applications in adsorption, enzyme stabilization and particularly drug delivery. These structures are MCM (Mobile Composition of Matter), SBA (Santa Barbara Amorphous), TUD (Technische Universiteit Delft), HMS (Hollow Mesoporous Silica) and MCF (Meso Cellular Form) (Yue et al., 2000; Heikkilä et al., 2007; Martínez-Edo et al., 2018; Wang et al., 2018).

### Mobile Composition of Matter

**FIGURE 3:**
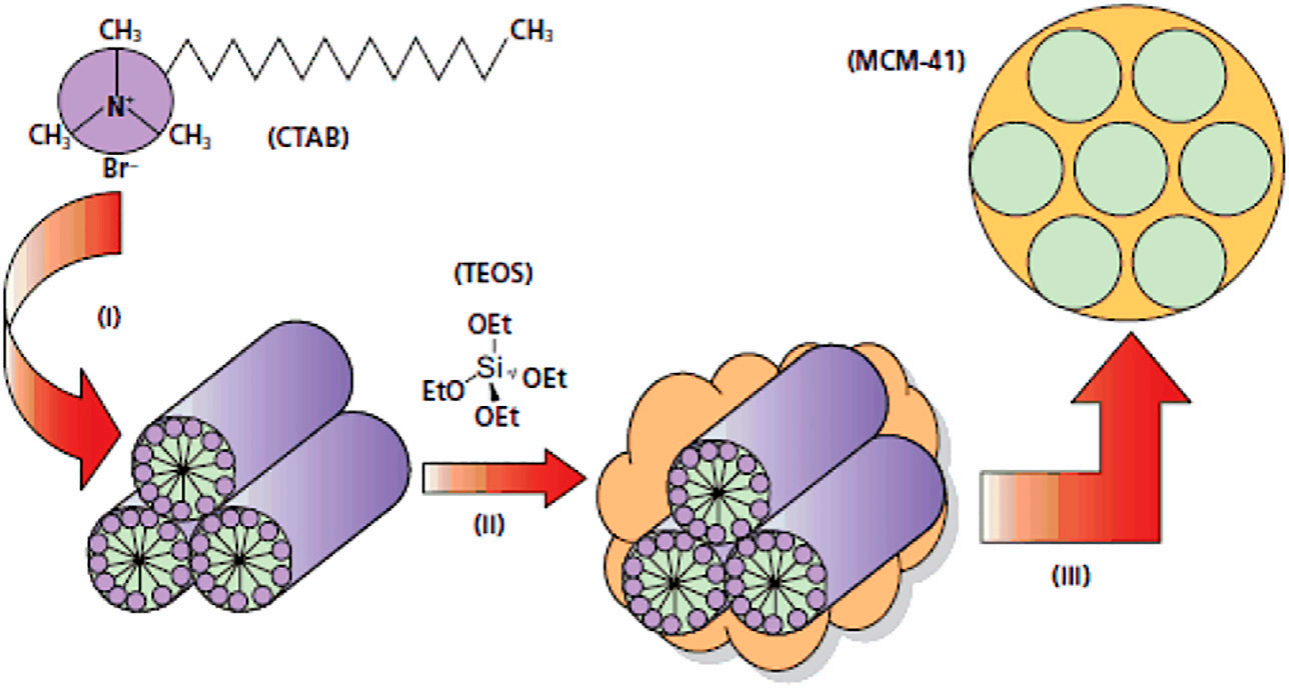
Synthesis of MCM-41 in basic media (Janicke et al., 1999).

These adsorbents are the first material in the mesopores generation that were synthesized in1992, which was the first step in the design of novel silica meso-carriers (Vadia and Rajput, 2011). The two groups identified from this family are MCM-41 (Chen et al., 1993) and MCM-48 (Kim et al., 2010). The synthesis of these materials is based on the creation of a liquid crystalline mesophase of surfactants that takes place in an acidic or base media (Janicke et al., 1999). Their appearance, shape, and pore size can be changed by manipulating pH and adding co-solvent. During the synthesis of MCM, from CTAB (Cethyl trimethyl ammonium Bromide) cationic surfactant, which is strongly stirred in a high-temperature in a basic solution are apply. TEOS is then added and the resulting solution is heated at high temperature to be stirred for 2 h. After the reaction is complete, the product is filtered, rinsed with water and ethanol and then dried under vacuum. The surfactant is then removed by acid wash (Figure 3) (Janicke et al., 1999).

Depending on the synthesis conditions, inorganic silica templates can be hexagonal, irregular, cubic, and so on. The first case was reported in the release of silica porous mesoporous to carriers of MCM-41 with ibuprofen (Salonen et al., 2008). Kavallaro et al. used Al Si- MCM-41 mesoporous silica for drugs such as difflonisal and its sodium salt and naproxen. Qiu and et al. investigated the Si-MCM-41 system as carrier of the drug captopril in water. They obtained a cavity size of 4.3 nm and a loading of 32.5%, while Kavrallo et al. obtained a cavity size of 2.79 nm and a total of 15.6% loading. The lower drug loading for Al Si-MCM-41 is presence of Al and Si metals of loaded in the MCM-41 pores, so if the size of the pore is less, the amount of drug loading is increases (Cavallaro et al., 2004).

Zeng et al. synthesized MCM-41 with the organic group of aminopropyl as carriers of aspirin and the results showed its release properties were influenced by the amount and distribution of aminopropyl groups in the pores wall and their regular structure (Zeng et al., 2005). MCM-48 is another mesoporous carrier from MCM group that has the three-dimensional shape group and cubic cavities, and the delayed release of the erythromycin antibiotic from this adsorbent was observed (Izquierdo-Barba et al., 2005). Lz-quierdo-Barba et al. conducted synthesis of MCM-48/erythromycin and MCM-48/ibuprofen nanocarriers. They illustrate that both have good release (Izquierdo-Barba et al., 2005). Also, these matters have been reported to increase the dissolution rate of piroxicam analgesic (Patil et al., 2019).

### Santa Barbara Amorphous

**FIGURE 4:**
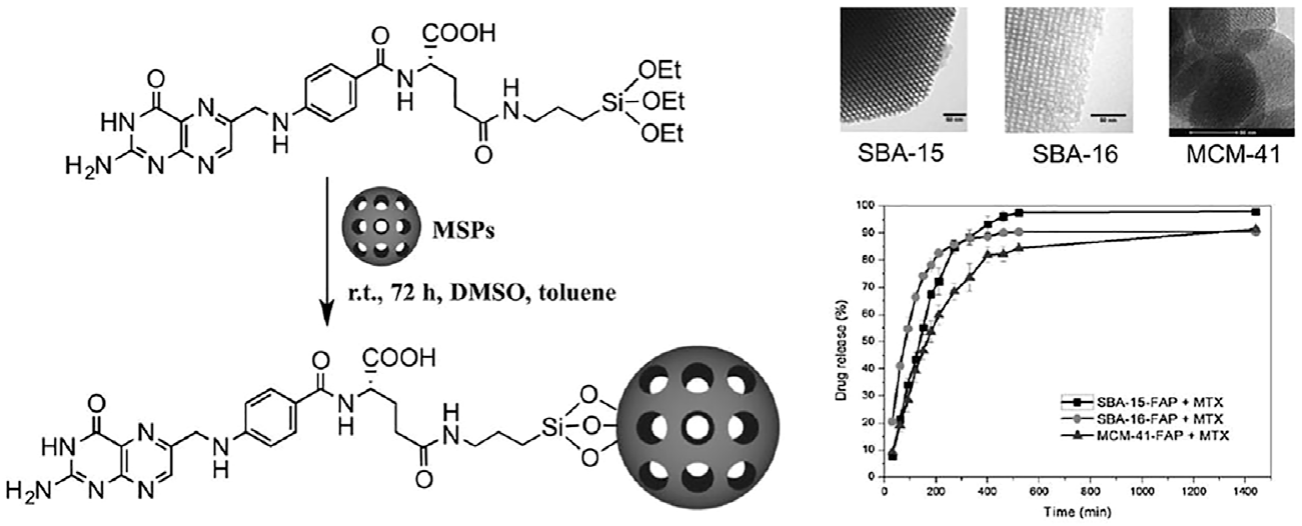
Loading of folic acid into SBA-15/NH_2_.

In1998, silica materials with regular meso pores synthesized in acidic conditions using non-ionic copolymers were synthesized with high amounts of polyethylene oxide and polypropylene oxide such as F-127 and P-123 pluronic (Speybroeck et al., 2009). The naming of these batches is based on their shape, such that the SBA-1 has a cage shape with cube circular pores (Che et al., 2001), SBA-11 (cubic) (Che et al., 2001), SBA-3 has a six-sided cylindrical porosity shape (Chen et al., 2004), SBA-14 (sheet) (Yu et al., 2018), SBA-15 has a two-dimensional hexagon shape (Margolese et al., 2000) and SBA-16 has a cubic cage structure (Rivera-Muñoz and Huirache-Acuña, 2010). Among these mesostructures, the SBA-15, followed by the SBA-16, quickly became the focus of attention. Because they have desirable surfaces, physicochemical properties such as low toxicity, biocompatibility, biodegradability, low-cost sources for synthesis, and are widespread their daily applications (Speybroeck et al., 2009). The SBA mesoporous pore wall is thicker than the MCM; although their specific surface area and pore volume are smaller than the MCM group, but they have a high mechanical and thermal stability (Speybroeck et al., 2009). Recently, the use of SBA-15 as a drug carrier has been evaluated and proven to have a structural shape and pore size effect on atenolol release. The appearance of the properties can be changed by altering the synthesis temperature. It also appears that SBA-15 is leading to the slow release of drugs (Speybroeck et al., 2009). Wang et al. showed that the choice of aqueous and non-aqueous solvents for loading into the SBA-15 had a significant effect on the rate of drug dissolution (Wang, 2009). An acceptable therapeutic target of hybrid porous mesoporous silica to which amine functional groups were added was also observed in folic acid loading (Figure 4) (Freitas et al., 2016).

Melaerts et al. locate itraconazole, a low-soluble antifungal drug in porous SBA-15, which increased its release. Zelenak et al. stabilized two Zn_3_(benzoate)_6_(nicotinamide)_2_ ZnNIA and Zn (benzoate)_2_(3-pyridinemethanol)_2_]_n_(ZnPCB) antibacterial drug complexes on SBA-16 and examined their release (Zeleňák et al., 2005).

### Other Silica Mesopores

TUD-1 is similar to foam because of its three-dimensional structure, where ibuprofen loading has led to improved drug release rates. Its synthesis conditions are slightly different from that of porous silica nanoparticles and the manufacturing process is carried out without the presence of surfactants, which is essential in terms of toxicity reduction and economic feasability. It has a high absorption capacity and is suitable for loading water-soluble drugs (Heikkilä et al., 2007). HMS has been reported as another meso-silica with a porous structure, the central part of which is hollow, which has given it the potential to load the drug as a nano-carrier (Zhu et al., 2005). The loading of ibuprofen and vancomycin by MCFs has been reported to have resulted in their dissolution rate increasing and as a good candidate for drug delivery (Zhu et al., 2009).

The incorporation of magnetic nanoparticles with mesoporous nanoparticles with a particle diameter of about 150 nm and pore size of 4 nm also showed slow drug release, similar to ibuprofen (Kim et al., 2006).

## Functionalization of Mesoporous Silica

Modification of the surface of the porous silica nanoparticles is carried out to enhance their different physical and chemical attributes. As mentioned, porous silica materials have an unusually broad surface area. Their surface is covered with silanol groups, which makes their cavities surface functional to be adjustable, which dramatically enhances the various physical and chemical properties (Kim et al., 2006). For example, the mesoporous surface of the silica can be modified by sulfonic acid groups derived from oxidized mercapto. Modified mesosilica can also be obtained with the aldehyde functional group by reaction with the amine and glutaraldehyde functional groups. Therefore, the various groups are capable of generating internal reactions such as hydrogen bonding, electrostatic adsorption, and covalent bonding with host molecules. Also, an organoalkoxy silane can be replaced with the cyano group present in the mesoporous surface, and the modified groups in the presence of sulfuric acid as catalysts were variated through hydrolysis by acidic groups.

### Post-synthesis Grafting

**FIGURE 5:**
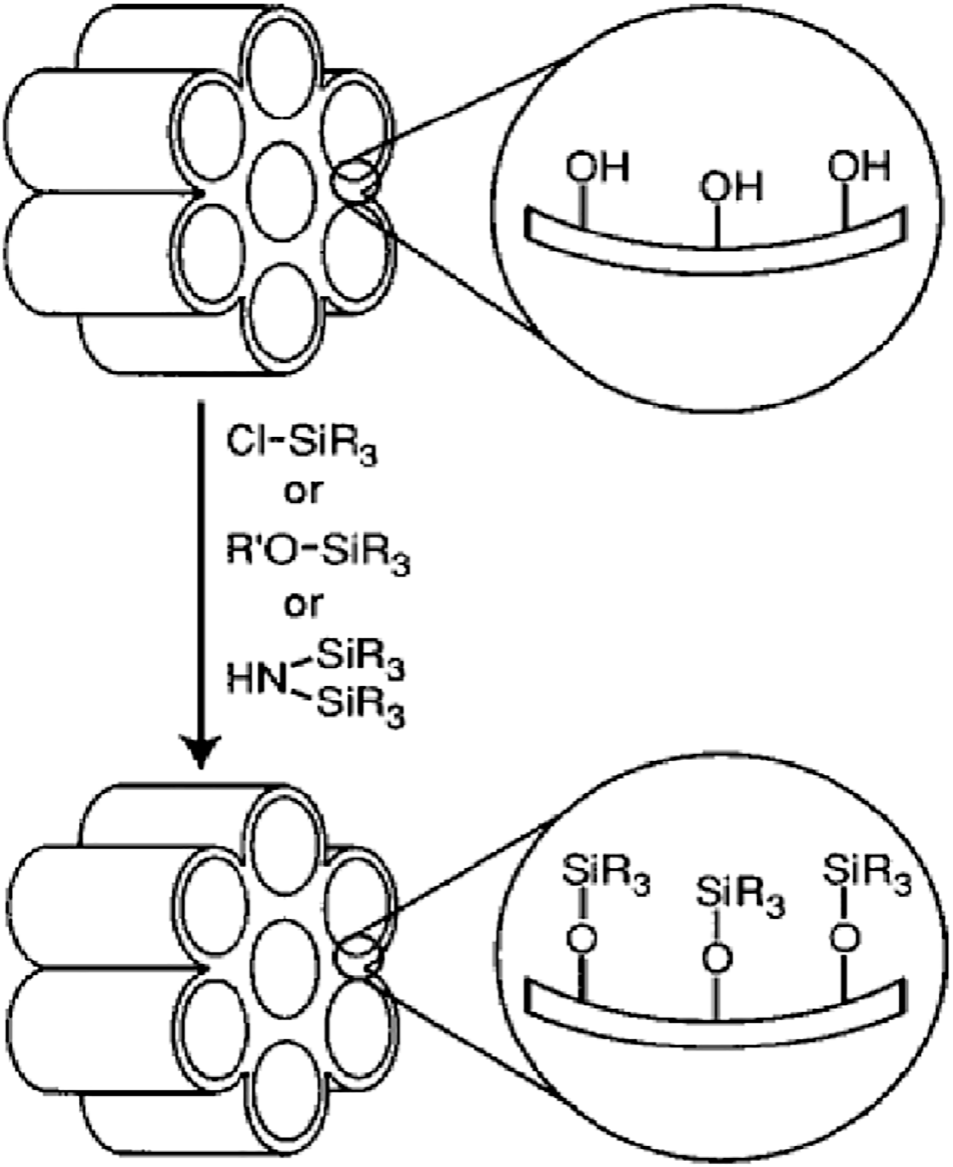
Functionalization of mesoporous silica by grafting.

Grafting is a type of post-fabrication process used to modify the surface of pre-fabricated porous silica material which bonds the functional groups connected to their surface after removing the surfactant (Zhang et al., 2004; Lesaint et al., 2005). The abundant silanol groups present on the surface of the silica porous material are applied as suitable junctions for functionalization with organic functional groups (Figure 5) (Barczak, 2019).

After removal of the surfactant, the organosilanes can be attached to the silanol channels using trichloro organosilanes or trialkoxy organosilanes (Barczak, 2019). A series of organosilanes including amine (Aguado et al., 2009), thiol (Li et al., 2011), chloride (Han et al., 2003), cyano (Prouzet et al., 1999), ester (Prouzet et al., 1999), aldehyde (Yin et al., 2020), epoxy (Yeganeh et al., 2019), anhydride (Park et al., 2019), isocyanate (Ratirotjanakul et al., 2019), phosphor (Yamada et al., 2019), imidazole (Nuri et al., 2019), ammonium (Lagarde et al., 2019), acryl (Xiao Song et al., 2019), alkyl (Nuntang et al., 2019), and phenyl (Gao et al., 2019) are available for application in the grafting procedure. The grafting is done by silicification so that the reaction is carried out on free (Si-OH) and attached silanol (HO-Si-OH) groups (Barczak, 2019). To diversify the surface or cavity walls of silica nanoparticles, silanol groups bonded hydrogen have limited access because they form hydrophilic networks between themselves (Barczak, 2019). It is worth noting that the main structure of the mesoporous does not change after applying the grafting procedure (Zhang et al., 2004). Silica mesostructures have two inner and exterior parts. Functional groups on the outer part will be more accessible than the inner part but the presence of these patches will diminish the use of pores space (Lesaint et al., 2005). To minimize the barriers of the outer surface in reaction and selective optimization, the outer surfaces may be coated to reduce chemical reactions before the functionalization of the inner surfaces occurs (Zhang et al., 2004; Lesaint et al., 2005). It is also possible to modify the surface in a controlled manner through site-selective grafting methods. Thus, in the first step, the cavities are filled with surfactants, and the exterior modification is accomplished by the proximity of the template to a solution such as a trimethylsilyl chloride (Lesaint et al., 2005). Then, template and finally the interior pore surfaces, for example, are functionalized with the phenyl propyl dimethyl chlorosilane sample (Zhang et al., 2004).

### Co-Condensation

The co-condensation matter is, in fact, sol-gel chemistry between tetraalkoxysilanes and one or more of the organoalkoxy silanes with Si-C bonds (Gao et al., 2019). In its mechanism of operation, silica precursor with a organotrialkoxy silanes (R^/^(Si(OR)_3_) precursor in neutral, acidic media that the organosilane precursor plays two important roles in silica-based skeletal formation and binding of an organic group to the skeleton (Hamoudi and Kaliaguine, 2002). Due to the presence of organic groups in the synthesized nanoparticles, template removal should be performed under appropriate conditions in terms of temperature and pH (Wu et al., 2013). Template removal is generally preferred by chemical extraction, and depending on the nature of the template and the manufacturing process, chemical extraction of the surfactant molecules is carried out by refluxing with ethanol or ethanol/HCl under highly acidic H_2_SO_4_ conditions. Functional groups such as polypropylene glycol, trimethylbenzene and tetradecane can also be used to achieve modified ordered mesoporous (Diagboya and Dikio, 2018). One of the advantages of this method, compared to the post-synthesis grafting, is the applicability of a wide range of organoalkoxy silanes (Jeelani et al., 2020) suitable for a wide range of reaction conditions (Fukuda and Yoshitake, 2019), homogeneous coverage of functional groups (Putz et al., 2019), and high loading of functional groups without destructing the regular structure of the pores (Nguyen et al., 2019).

## Silica Mesoporous for Drug Delivery

The use of nanosilica materials for controlled drug delivery and release has been reported since the beginning of 1983 (He and Shi, 2011). To date, silica nanoparticles are widely used as drug carriers due to their easy compatibility and formulation with drugs (Yamada et al., 2019). Advantages of using mesoporous silica to transport bioactive molecules include protecting cargo from physiological destruction of controlled release of drugs (Tang et al., 2012), longer durability in the bloodstream (Privett et al., 2011), improving targeted drug delivery (Wang et al., 2010), and reducuction of side effects to healthy tissues (Zhou et al., 2017). In recently years, non-steroidal Analgesics (NSA) have been considered. Non-steroidal painkillers, which are analgesic (Aiello et al., 2002), fever-inducing (N et al., 2019), and platelet-inhibiting (Brogan et al., 2019), are one of the most widely used medical drugs whose biological target is the cyclooxygenase (COX) enzyme (Voiriot et al., 2019). These drugs actually suppress prostaglandins, which are chemical messengers for pain, fever, and inflammation (Mahalanobish et al., 2019). Their strategy of action is to inactivate the performance of the COX, which has the potential to convert fatty acid to prostaglandin, or in other words, overcome the production of prostaglandin by COX (FitzGerald, 2003). In addition to the advantages of these drugs, they have side effects, including excessive body retention (Marsh et al., 1972) and low solubility (Rives et al., 2013), which disrupt the treatment. As a result, in recent years, silica porous nanoparticles have been proposed to address these problems. Increasing the solubility of NSA drugs can be justified by the size of the cavities (Mellaerts et al., 2008), easy dispersion, indirect transfer of the drug into the aqueous media (Li et al., 2007), utilization of surfactants (Chen et al., 2010), and long durability in the body by biodegradable coatings such as polyethylene glycol (PEG) (Morelli et al., 2011). Research has reported the release of a number of drugs such as ibuprofen (Charnay et al., 2004), diclofenac (Barczak, 2018), melasamine (Mes) (Tiwari et al., 2019), naproxen (Halamová et al., 2010), piroxicam (Ambrogi et al., 2007), celecoxib (Zhao et al., 2012) and mefenamic acid (Mustafa and Hodali, 2015) and etc.

Yoncheva et al. synthesized MCM-41/carbopol/indomethacine nanocarrier. They reported that this meso-carrier showed little cytotoxicity due to carbopol coating (Tzankov et al., 2013). Naproxen (Nap) is a potent Cox enzyme inhibitor that reduces prostaglandin production and exhibits analgesic and anti-inflammatory effects (Lejal et al., 2013). Therefore, Halamova et al. two mesostructures used amine- non-amine functionalized hexagonal MCM-41 to release Nap. Comparison of these two carriers showed that after 72 h, the efficiency of release of MCM-41/Nap was 95% and with MCM-41/amine/Nap 90%, respectively. Their groups’ research also showed that the release of the Nap using SBA-15 with a larger cavity size was less than MCM-41 with a smaller cavity size (Halamová and Zeleňák, 2012). The preparation of SBA-15 decorated by glycidyl methacrylate (GMA) as nanocarrier for release of ibuprofen and Mes was carried out by Rehman and coworkers. Copper was modified with hydrophobic ligands to slow and adjust the release of the two drugs and increase the ibuprofen and Mes- SBA-15 interaction. The impact of pH in their research revealed interesting results. The *in vitro* release rate from the functionalized SBA-15 was slow in simulated gastric fluid where pH = 1.2 was less than 10% of Mes and ibuprofen was released in initial time 8 h, while comparatively high release rates were observed in simulated intestinal (pH = 6.8) and simulated body fluids (pH = 7.2) (Rehman et al., 2016).

## Conclusion and Clinical Translation

MSNs are one-of-a-kind nanoparticles that combine the chemical and physical stability of silica with the potential of the mesoporous structure’s network of cavities. MSNs’ unique properties, such as pore-volume, their great loading capability, their controllable particle size and shape, and their high drug loading capacity, make them an excellent carrier for nano-drug delivery systems. MSNs can be anchored with many polymers, proteins, enzymes and due to their ease of functionalization, make them a good candidate for drug delivery applications.

Standardization of manufacturing techniques is vital for achieving reproducibility in MSN synthesis. It is also critical that the generated nanoparticles have the proper stability, dispersibility; and any surface functionalization method should be standardized before reaching the clinic. More importantly, additional MSN biodistribution studies on various animal models should be conducted to be certain of the MSNs’ ultimate fate. From a broad view, it is clear that significant progress has been made in the design and development of MSNs for biological applications. However, much more work needs to be done before clinical translation can be accomplished.

Based on their efficacy in clinical studies, silica nanoparticles are developing as a viable diagnostic and delivery platform, and might play a crucial role in the development of next-generation theranostics, nanovaccines, and formulations to orally transport peptides and proteins. However, establishing safety from chronic exposure, establishing long-term toxicological profiles from various routes of administration, investigating reliable scale-up methods, and synthesizing reproducible silica nanoparticles with minimal batch-to-batch variation, are all major obstacles that must be overcome before silica nanoparticles can be used in clinical trials. Furthermore, only solid silica nanoparticles with no or small pores have been clinically evaluated to date. These nanoparticles have a low cargo-loading capacity which is particularly problematic for nucleic-acid-based medicines.

## References

[B1] Aguado J. Arsuaga J. M. Arencibia A. Lindo M. Gascón V. (2009). Aqueous Heavy Metals Removal by Adsorption on Amine-Functionalized Mesoporous Silica. J. Hazard. Mater. 163, 213–221. 10.1016/j.jhazmat.2008.06.080 18675509

[B2] Ahmed T. Aljaeid B. (2016). Preparation, Characterization, and Potential Application of Chitosan, Chitosan Derivatives, and Chitosan Metal Nanoparticles in Pharmaceutical Drug Delivery. Dddt 10, 483. 10.2147/dddt.s99651 26869768 PMC4734734

[B3] Aiello R. Cavallaro G. Giammona G. Pasqua L. Pierro P. Testa F. (2002). “Mesoporous Silicate as Matrix for Drug Delivery Systems of Non-steroidal Antinflammatory Drugs,” in Studies in Surface Science and Catalysis (Elsevier), 1165–1172. 10.1016/s0167-2991(02)80276-2

[B4] Ambrogi V. Perioli L. Marmottini F. Giovagnoli S. Esposito M. Rossi C. (2007). Improvement of Dissolution Rate of Piroxicam by Inclusion into MCM-41 Mesoporous Silicate. Eur. J. Pharm. Sci. 32, 216–222. 10.1016/j.ejps.2007.07.005 17826966

[B5] Ang C. W. Tan L. Qu Z. West N. P. Cooper M. A. Popat A. (2021). Mesoporous Silica Nanoparticles Improve Oral Delivery of Antitubercular Bicyclic Nitroimidazoles. Washington, DC: ACS biomaterials science & engineering. 10.1021/acsbiomaterials.1c00807 PMC9554870 34464089

[B6] Anglin E. Cheng L. Freeman W. Sailor M. (2008). Porous Silicon in Drug Delivery Devices and Materials☆. Adv. Drug Deliv. Rev. 60, 1266–1277. 10.1016/j.addr.2008.03.017 18508154 PMC2710886

[B7] Banerjee A. Qi J. Gogoi R. Wong J. Mitragotri S. (2016). Role of Nanoparticle Size, Shape and Surface Chemistry in Oral Drug Delivery. J. Controlled Release 238, 176–185. 10.1016/j.jconrel.2016.07.051 PMC5289391 27480450

[B8] Barczak M. (2019). Functionalization of Mesoporous Silica Surface with Carboxylic Groups by Meldrum's Acid and its Application for Sorption of Proteins. J. Porous Mater. 26, 291–300. 10.1007/s10934-018-0655-7

[B9] Barczak M. (2018). Synthesis and Structure of Pyridine-Functionalized Mesoporous SBA-15 Organosilicas and Their Application for Sorption of Diclofenac. J. Solid State. Chem. 258, 232–242. 10.1016/j.jssc.2017.10.006

[B10] Bhatia S. (2016). “Nanoparticles Types, Classification, Characterization, Fabrication Methods and Drug Delivery Applications,” in Natural Polymer Drug Delivery Systems (Springer), 33–93. 10.1007/978-3-319-41129-3_2

[B11] Brogan S. E. Mandyam S. Odell D. W. (2019). “Nonopioid Analgesics,” in Pharmacology and Physiology for Anesthesia (Elsevier), 369–389. 10.1016/b978-0-323-48110-6.00019-3

[B12] Cavallaro G. Pierro P. Palumbo F. S. Testa F. Pasqua L. Aiello R. (2004). Drug Delivery Devices Based on Mesoporous Silicate. Drug Deliv. 11, 41–46. 10.1080/10717540490265252 15168790

[B13] Charnay C. Bégu S. Tourné-Péteilh C. Nicole L. Lerner D. A. Devoisselle J. M. (2004). Inclusion of Ibuprofen in Mesoporous Templated Silica: Drug Loading and Release Property. Eur. J. pharmaceutics biopharmaceutics 57, 533–540. 10.1016/j.ejpb.2003.12.007 15093603

[B14] Che S. Sakamoto Y. Terasaki O. Tatsumi T. (2001). Control of crystal Morphology of SBA-1 Mesoporous Silica. Chem. Mater. 13, 2237–2239. 10.1021/cm010297f

[B15] Chen C.-Y. Li H.-X. Davis M. E. (1993). Studies on Mesoporous Materials. Microporous Mater. 2, 17–26. 10.1016/0927-6513(93)80058-3

[B16] Chen F.-H. Zhang L.-M. Chen Q.-T. Zhang Y. Zhang Z.-J. (2010). Synthesis of a Novel Magnetic Drug Delivery System Composed of Doxorubicin-Conjugated Fe3O4 Nanoparticle Cores and a PEG-Functionalized Porous Silica Shell. Chem. Commun. 46, 8633–8635. 10.1039/c0cc02577a 20941412

[B17] Chen F. Xu X.-J. Shen S. Kawi S. Hidajat K. (2004). Microporosity of SBA-3 Mesoporous Molecular Sieves. Microporous mesoporous Mater. 75, 231–235. 10.1016/j.micromeso.2004.07.028

[B18] Chen M. Hu J. Wang L. Li Y. Zhu C. Chen C. (2020). Targeted and Redox-Responsive Drug Delivery Systems Based on Carbonic Anhydrase IX-Decorated Mesoporous Silica Nanoparticles for Cancer Therapy. Sci. Rep. 10, 14447–14512. 10.1038/s41598-020-71071-1 32879359 PMC7467921

[B19] Chen X. J. Zhang X. Q. Liu Q. Zhang J. Zhou G. (2018). Nanotechnology: a Promising Method for Oral Cancer Detection and Diagnosis. J. Nanobiotechnology 16, 52–17. 10.1186/s12951-018-0378-6 29890977 PMC5994839

[B20] Cooley M. Sarode A. Hoore M. Fedosov D. A. Mitragotri S. Sen Gupta A. (2018). Influence of Particle Size and Shape on Their Margination and wall-adhesion: Implications in Drug Delivery Vehicle Design across Nano-To-Micro Scale. Nanoscale 10, 15350–15364. 10.1039/c8nr04042g 30080212 PMC6247903

[B21] Diagboya P. N. E. Dikio E. D. (2018). Silica-based Mesoporous Materials; Emerging Designer Adsorbents for Aqueous Pollutants Removal and Water Treatment. Microporous Mesoporous Mater. 266, 252–267. 10.1016/j.micromeso.2018.03.008

[B22] Djekic L. Martinović M. Dobričić V. Čalija B. Medarević Đ. Primorac M. (2019). Comparison of the Effect of Bioadhesive Polymers on Stability and Drug Release Kinetics of Biocompatible Hydrogels for Topical Application of Ibuprofen. J. Pharm. Sci. 108, 1326–1333. 10.1016/j.xphs.2018.10.054 30395827

[B23] Dudarko О. А. Sliesarenko V. V. Tomin O. O. Pamin K. Serwicka E. M. Zub Y. L. (2019). “Mesoporous Surface-Functionalized Silicas and Their Application in Sorption and Catalysis,” in Biocompatible Hybrid Oxide Nanoparticles for Human Health (Elsevier), 51–65. 10.1016/b978-0-12-815875-3.00004-7

[B24] El-Boubbou K. (2018). Magnetic Iron Oxide Nanoparticles as Drug Carriers: Preparation, Conjugation and Delivery. Nanomedicine 13, 929–952. 10.2217/nnm-2017-0320 29546817

[B25] FitzGerald G. A. (2003). COX-2 and beyond: Approaches to Prostaglandin Inhibition in Human Disease. Nat. Rev. Drug Discov. 2, 879–890. 10.1038/nrd1225 14668809

[B26] Florek J. Caillard R. Kleitz F. (2017). Evaluation of Mesoporous Silica Nanoparticles for Oral Drug Delivery - Current Status and Perspective of MSNs Drug Carriers. Nanoscale 9, 15252–15277. 10.1039/c7nr05762h 28984885

[B27] Freitas L. B. d. O. Bravo I. J. G. Macedo W. A. d. A. de Sousa E. M. B. (2016). Mesoporous Silica Materials Functionalized with Folic Acid: Preparation, Characterization and Release Profile Study with Methotrexate. J. Sol-gel Sci. Technol. 77, 186–204. 10.1007/s10971-015-3844-8

[B28] Fukuda K. Yoshitake H. (2019). Alternating Copolymerization of Functionalized Silica Nanoparticles. Polymer 165, 133–141. 10.1016/j.polymer.2019.01.036

[B29] Gao P. Liang Z. Zhao Z. Wang W. Yang C. Hu B. (2019). Enhanced Adsorption of Steroid Estrogens by One-Pot Synthesized Phenyl-Modified Mesoporous Silica: Dependence on Phenyl-Organosilane Precursors and pH Condition. Chemosphere 234, 438–449. 10.1016/j.chemosphere.2019.06.089 31228846

[B30] Goetjen T. A. Liu J. Wu Y. Sui J. Zhang X. Hupp J. T. (2020). Metal-organic Framework (MOF) Materials as Polymerization Catalysts: a Review and Recent Advances. Chem. Commun. 56, 10409–10418. 10.1039/d0cc03790g 32745156

[B31] Guha A. Biswas N. Bhattacharjee K. Sahoo N. Kuotsu K. (2016). pH Responsive Cylindrical MSN for Oral Delivery of Insulin-Design, Fabrication and Evaluation. Drug Deliv. 23, 3552–3561. 10.1080/10717544.2016.1209796 27540687

[B32] Halamová D. Badaničová M. Zeleňák V. Gondová T. Vainio U. (2010). Naproxen Drug Delivery Using Periodic Mesoporous Silica SBA-15. Appl. Surf. Sci. 256, 6489–6494. 10.1016/j.apsusc.2010.04.044

[B33] Halamová D. Zeleňák V. (2012). NSAID Naproxen in Mesoporous Matrix MCM-41: Drug Uptake and Release Properties. J. Incl Phenom Macrocycl Chem. 72, 15–23. 10.1007/s10847-011-9990-x

[B34] Hamoudi S. Kaliaguine S. (2002). Periodic Mesoporous Organosilica from Micellar Oligomer Template solutionElectronic Supplementary Information (ESI). Chem. Commun., 2118–2119. 10.1039/b207134g 12357806

[B35] Han S. Hou W. Dang W. Xu J. Hu J. Li D. (2003). Synthesis of Rod-like Mesoporous Silica Using Mixed Surfactants of Cetyltrimethylammonium Bromide and Cetyltrimethylammonium Chloride as Templates. Mater. Lett. 57, 4520–4524. 10.1016/s0167-577x(03)00355-0

[B36] He Q. Shi J. (2011). Mesoporous Silica Nanoparticle Based Nano Drug Delivery Systems: Synthesis, Controlled Drug Release and Delivery, Pharmacokinetics and Biocompatibility. J. Mater. Chem. 21, 5845–5855. 10.1039/c0jm03851b

[B37] Heikkilä T. Salonen J. Tuura J. Hamdy M. Mul G. Kumar N. (2007). Mesoporous Silica Material TUD-1 as a Drug Delivery System. Int. J. pharmaceutics 331, 133–138. 10.1016/j.ijpharm.2006.09.019 17046183

[B38] Hughes G. A. (2005). Nanostructure-mediated Drug Delivery. Nanomedicine: nanotechnology, Biol. Med. 1, 22–30. 10.1016/j.nano.2004.11.009 17292054

[B39] Izquierdo-Barba I. Martinez Á. Doadrio A. L. Pérez-Pariente J. Vallet-Regí M. (2005). Release Evaluation of Drugs from Ordered Three-Dimensional Silica Structures. Eur. J. Pharm. Sci. 26, 365–373. 10.1016/j.ejps.2005.06.009 16185852

[B40] Jahangirian H. Ghasemian lemraski E. Webster T. J. Rafiee-Moghaddam R. Abdollahi Y. (2017). A Review of Drug Delivery Systems Based on Nanotechnology and green Chemistry: green Nanomedicine. Ijn Vol. 12, 2957–2978. 10.2147/ijn.s127683 PMC5396976 28442906

[B41] Janicke M. T. Landry C. C. Christiansen S. C. Birtalan S. Stucky G. D. Chmelka B. F. (1999). Low Silica MCM-41 Composites and Mesoporous Solids. Chem. Mater. 11, 1342–1351. 10.1021/cm981135v

[B42] Janjua T. I. Ahmed-Cox A. Meka A. K. Mansfeld F. M. Forgham H. Ignacio R. M. C. (2021). Facile Synthesis of Lactoferrin Conjugated Ultra Small Large Pore Silica Nanoparticles for the Treatment of Glioblastoma. Nanoscale 13, 16909–16922. 10.1039/d1nr03553c 34533167

[B43] Jeelani P. G. Mulay P. Venkat R. Ramalingam C. (2020). Multifaceted Application of Silica Nanoparticles. A Review. Silicon 12, 1337–1354. 10.1007/s12633-019-00229-y

[B44] Jindal A. Juneja S. Bakshi M. Chaudhuri P. Bhattacharya J. (2019). Mesoporous Zinc Silicate Bio-Composite: Preparation, Characterization and *In Vitro* Evaluation. Microporous Mesoporous Mater. 277, 124–131. 10.1016/j.micromeso.2018.10.025

[B45] Kamaly N. Yameen B. Wu J. Farokhzad O. C. (2016). Degradable Controlled-Release Polymers and Polymeric Nanoparticles: Mechanisms of Controlling Drug Release. Chem. Rev. 116, 2602–2663. 10.1021/acs.chemrev.5b00346 26854975 PMC5509216

[B46] Kapoor D. N. Bhatia A. Kaur R. Sharma R. Kaur G. Dhawan S. (2015). PLGA: a Unique Polymer for Drug Delivery. Ther. Deliv. 6, 41–58. 10.4155/tde.14.91 25565440

[B47] Khdair A. Hamad I. Alkhatib H. Bustanji Y. Mohammad M. Tayem R. (2016). Modified-chitosan Nanoparticles: Novel Drug Delivery Systems Improve Oral Bioavailability of Doxorubicin. Eur. J. Pharm. Sci. 93, 38–44. 10.1016/j.ejps.2016.07.012 27473308

[B48] Kim J. Lee J. E. Lee J. Yu J. H. Kim B. C. An K. (2006). Magnetic Fluorescent Delivery Vehicle Using Uniform Mesoporous Silica Spheres Embedded with Monodisperse Magnetic and Semiconductor Nanocrystals. J. Am. Chem. Soc. 128, 688–689. 10.1021/ja0565875 16417336

[B49] Kim J. M. Han Y.-J. Stucky G. D. Chmelka B. F. (2000). One-step Synthesis of Ordered Mesocomposites with Non-ionic Amphiphilic Block Copolymers: Implications of Isoelectric point, Hydrolysis Rate and Fluoride. Chem. Commun., 2437–2438. 10.1039/b005608l

[B50] Kim T.-W. Chung P.-W. Lin V. S.-Y. (2010). Facile Synthesis of Monodisperse Spherical MCM-48 Mesoporous Silica Nanoparticles with Controlled Particle Size. Chem. Mater. 22, 5093–5104. 10.1021/cm1017344

[B51] Lagarde F. Srour H. Berthet N. Oueslati N. Bousquet B. Nunes A. (2019). Investigating the Role of SBA-15 Silica on the Activity of Quaternary Ammonium Halides in the Coupling of Epoxides and CO2. J. CO2 Utilization 34, 34–39. 10.1016/j.jcou.2019.05.023

[B52] Lee C.-H. Lo L.-W. Mou C.-Y. Yang C.-S. (2008). Synthesis and Characterization of Positive-Charge Functionalized Mesoporous Silica Nanoparticles for Oral Drug Delivery of an Anti-inflammatory Drug. Adv. Funct. Mater. 18, 3283–3292. 10.1002/adfm.200800521

[B53] Lejal N. Tarus B. Bouguyon E. Chenavas S. Bertho N. Delmas B. (2013). Structure-based Discovery of the Novel Antiviral Properties of Naproxen against the Nucleoprotein of Influenza A Virus. Antimicrob. Agents Chemother. 57, 2231–2242. 10.1128/aac.02335-12 23459490 PMC3632891

[B54] Lesaint C. Frébault F. Delacôte C. Lebeau B. Marichal C. Walcarius A. (2005). “Synthesis and Characterization of Mesoporous Silicas Functionalized by Thiol Groups, and Application as Sorbents for Mercury (II),” in Studies in Surface Science and Catalysis (Elsevier), 925–932. 10.1016/s0167-2991(05)80305-2

[B55] Li G. Zhao Z. Liu J. Jiang G. (2011). Effective Heavy Metal Removal from Aqueous Systems by Thiol Functionalized Magnetic Mesoporous Silica. J. Hazard. Mater. 192, 277–283. 10.1016/j.jhazmat.2011.05.015 21616588

[B56] Li X.-Y. Song Y. Zhang C.-X. Zhao C.-X. He C. (2021). Inverse CO2/C2H2 Separation in a Pillared-Layer Framework Featuring a Chlorine-Modified Channel by Quadrupole-Moment Sieving. Sep. Purif. Tech. 279.119608. 10.1016/j.seppur.2021.119608

[B57] Li X. Zhang L. Dong X. Liang J. Shi J. (2007). Preparation of Mesoporous Calcium Doped Silica Spheres with Narrow Size Dispersion and Their Drug Loading and Degradation Behavior. Microporous Mesoporous Mater. 102, 151–158. 10.1016/j.micromeso.2006.12.048

[B58] Mahalanobish S. Saha S. Dutta S. Ghosh S. Sil P. C. (2019). “Anti-inflammatory Efficacy of Some Potentially Bioactive Natural Products against Rheumatoid Arthritis,” in Discovery and Development of Anti-inflammatory Agents from Natural Products (Elsevier), 61–100. 10.1016/b978-0-12-816992-6.00003-6

[B59] Manzano M. Vallet-Regí M. (2019). Ultrasound Responsive Mesoporous Silica Nanoparticles for Biomedical Applications. Chem. Commun. 55, 2731–2740. 10.1039/c8cc09389j PMC6667338 30694270

[B60] Margolese D. Melero J. A. Christiansen S. C. Chmelka B. F. Stucky G. D. (2000). Direct Syntheses of Ordered SBA-15 Mesoporous Silica Containing Sulfonic Acid Groups. Chem. Mater. 12, 2448–2459. 10.1021/cm0010304

[B61] Marsh A. Kim D. N. Lee K. T. Reiner J. M. Thomas W. A. (1972). Cholesterol Turnover, Synthesis, and Retention in Hypercholesterolemic Growing Swine. J. lipid Res. 13, 600–615. 10.1016/s0022-2275(20)39366-4 5075507

[B62] Martínez-Edo G. Balmori A. Pontón I. Martí del Rio A. Sánchez-García D. (2018). Functionalized Ordered Mesoporous Silicas (MCM-41): Synthesis and Applications in Catalysis. Catalysts 8, 617. 10.3390/catal8120617

[B63] Martínez-Ortega L. Mira A. Fernandez-Carvajal A. Mateo C. R. Mallavia R. Falco A. (2019). Development of a New Delivery System Based on Drug-Loadable Electrospun Nanofibers for Psoriasis Treatment. Pharmaceutics 11, 14. 10.3390/pharmaceutics11010014 PMC6359116 30621136

[B64] Martínez-Vázquez F. J. Cabañas M. V. Paris J. L. Lozano D. Vallet-Regí M. (2015). Fabrication of Novel Si-Doped Hydroxyapatite/gelatine Scaffolds by Rapid Prototyping for Drug Delivery and Bone Regeneration. Acta Biomater. 15, 200–209. 10.1016/j.actbio.2014.12.021 25560614

[B65] McClements D. J. (2018). Encapsulation, protection, and Delivery of Bioactive Proteins and Peptides Using Nanoparticle and Microparticle Systems: A Review. Adv. Colloid Interf. Sci. 253, 1–22. 10.1016/j.cis.2018.02.002 29478671

[B66] Meka A. Jenkins L. Dàvalos-Salas M. Pujara N. Wong K. Kumeria T. (2018). Enhanced Solubility, Permeability and Anticancer Activity of Vorinostat Using Tailored Mesoporous Silica Nanoparticles. Pharmaceutics 10, 283. 10.3390/pharmaceutics10040283 PMC6321298 30562958

[B67] Mellaerts R. Mols R. Jammaer J. A. G. Aerts C. A. Annaert P. Van Humbeeck J. (2008). Increasing the Oral Bioavailability of the Poorly Water Soluble Drug Itraconazole with Ordered Mesoporous Silica. Eur. J. Pharmaceutics Biopharmaceutics 69, 223–230. 10.1016/j.ejpb.2007.11.006 18164930

[B68] Meynen V. Cool P. Vansant E. F. (2009). Verified Syntheses of Mesoporous Materials. Microporous mesoporous Mater. 125, 170–223. 10.1016/j.micromeso.2009.03.046

[B69] Morelli C. Maris P. Sisci D. Perrotta E. Brunelli E. Perrotta I. (2011). PEG-templated Mesoporous Silica Nanoparticles Exclusively Target Cancer Cells. Nanoscale 3, 3198–3207. 10.1039/c1nr10253b 21725561

[B70] Mustafa F. M. Hodali H. A. (2015). “Use of Mesoporous Silicate Nanoparticles as Drug Carrier for Mefenamic Acid,” IOP Conf. Ser. Mater. Sci. Eng., 92, 012018. 10.1088/1757-899x/92/1/012018

[B71] N P. Ss A. Pv M. (2019). Comprehensive Biology of Antipyretic Pathways. Cytokine 116, 120–127. 10.1016/j.cyto.2019.01.008 30711851

[B72] Nairi V. Medda S. Piludu M. Casula M. F. Vallet-Regì M. Monduzzi M. (2018). Interactions between Bovine Serum Albumin and Mesoporous Silica Nanoparticles Functionalized with Biopolymers. Chem. Eng. J. 340, 42–50. 10.1016/j.cej.2018.01.011

[B73] Nguyen T. L. Choi Y. Kim J. (2019). Mesoporous Silica as a Versatile Platform for Cancer Immunotherapy. Adv. Mater. 31, 1803953. 10.1002/adma.201803953 30417454

[B74] Nuntang S. Yousatit S. Yokoi T. Ngamcharussrivichai C. (2019). Tunable Mesoporosity and Hydrophobicity of Natural Rubber/hexagonal Mesoporous Silica Nanocomposites. Microporous Mesoporous Mater. 275, 235–243. 10.1016/j.micromeso.2018.09.004

[B75] Nuri A. Mansoori Y. Bezaatpour A. (2019). N‐heterocyclic carbene-Palladium(II) Complex Supported on Magnetic Mesoporous Silica for Heck Cross‐coupling Reaction. Appl. Organometal Chem. 33, e4904. 10.1002/aoc.4904

[B76] Park J. Park S. S. Jo N.-J. Ha C.-S. (2019). Folic Acid-Polyethyleneimine Functionalized Mesoporous Silica Nanoparticles as a Controlled Release Nanocarrier. j nanosci nanotechnol 19, 6217–6224. 10.1166/jnn.2019.17054 31026940

[B77] Patil L. D. Verma U. Patil U. D. Naik J. B. Narkhede J. S. (2019). Inclusion of Aceclofenac in Mesoporous Silica Nanoparticles: Drug Release Study and Statistical Optimization of Encapsulation Efficiency by Response Surface Methodology. Mater. Tech. 34, 751–763. 10.1080/10667857.2019.1624301

[B78] Paunovska K. Gil C. J. Lokugamage M. P. Sago C. D. Sato M. Lando G. N. (2018). Analyzing 2000 *In Vivo* Drug Delivery Data Points Reveals Cholesterol Structure Impacts Nanoparticle Delivery. ACS nano 12, 8341–8349. 10.1021/acsnano.8b03640 30016076 PMC6115295

[B79] Privett B. J. Youn J. Hong S. A. Lee J. Han J. Shin J. H. (2011). Antibacterial Fluorinated Silica Colloid Superhydrophobic Surfaces. Langmuir 27, 9597–9601. 10.1021/la201801e 21718023 PMC3163484

[B80] Prouzet E. Cot F. Nabias G. Larbot A. Kooyman P. Pinnavaia T. J. (1999). Assembly of Mesoporous Silica Molecular Sieves Based on Nonionic Ethoxylated Sorbitan Esters as Structure Directors. Chem. Mater. 11, 1498–1503. 10.1021/cm9810281

[B81] Putz A.-M. Almásy L. Len A. Ianăşi C. (2019). Functionalized Silica Materials Synthesized *via* Co-condensation and post-grafting Methods. Fullerenes, Nanotubes and Carbon Nanostructures 27, 323–332. 10.1080/1536383x.2019.1593154

[B82] Ratirotjanakul W. Suteewong T. Polpanich D. Tangboriboonrat P. (2019). Amino Acid as a Biodegradation Accelerator of Mesoporous Silica Nanoparticles. Microporous Mesoporous Mater. 282, 243–251. 10.1016/j.micromeso.2019.02.033

[B83] Rehman F. Rahim A. Airoldi C. Volpe P. L. O. (2016). Preparation and Characterization of Glycidyl Methacrylate Organo Bridges Grafted Mesoporous Silica SBA-15 as Ibuprofen and Mesalamine Carrier for Controlled Release. Mater. Sci. Eng. C 59, 970–979. 10.1016/j.msec.2015.11.005 26652455

[B84] Reibaldi M. Avitabile T. Bandello F. Longo A. Bonfiglio V. Russo A. (2019). The Effectiveness of 0.6% Povidone Iodine Eye Drops in Reducing the Conjunctival Bacterial Load and Needle Contamination in Patients Undergoing Anti-VEGF Intravitreal Injection: a Prospective, Randomized Study. J. Clin. Med. 8, 1031. 10.3390/jcm8071031 PMC6678890 31337003

[B85] Rivera-Muñoz E. M. Huirache-Acuña R. (2010). Sol Gel-Derived SBA-16 Mesoporous Material. Ijms 11, 3069–3086. 10.3390/ijms11093069 20957080 PMC2956081

[B86] Rives V. Del Arco M. Martín C. (2013). Layered Double Hydroxides as Drug Carriers and for Controlled Release of Non-steroidal Antiinflammatory Drugs (NSAIDs): a Review. J. Controlled Release 169, 28–39. 10.1016/j.jconrel.2013.03.034 23583707

[B87] Salonen J. Kaukonen A. M. Hirvonen J. Lehto V.-P. (2008). Mesoporous Silicon in Drug Delivery Applications. J. Pharm. Sci. 97, 632–653. 10.1002/jps.20999 17546667

[B88] Sharma R. Kumar V. Kumar R. (2019). Distribution of Phytoliths in Plants: A Review. Geology. Ecology, Landscapes 3, 123–148. 10.1080/24749508.2018.1522838

[B89] Speybroeck M. V. Barillaro V. Thi T. D. Mellaerts R. Martens J. Humbeeck J. V. (2009). Ordered Mesoporous Silica Material SBA-15: a Broad-Spectrum Formulation Platform for Poorly Soluble Drugs. J. Pharm. Sci. 98, 2648–2658. 10.1002/jps.21638 19072861

[B90] Tandel H. Bhatt P. Jain K. Shahiwala A. Misra A. (2018). *In-Vitro* and *In-Vivo* Tools in Emerging Drug Delivery Scenario: Challenges and Updates. chapter, 1–24. 10.1201/b22448-1

[B91] Tang F. Li L. Chen D. (2012). Mesoporous Silica Nanoparticles: Synthesis, Biocompatibility and Drug Delivery. Adv. Mater. 24, 1504–1534. 10.1002/adma.201104763 22378538

[B92] Tarrahi R. Movafeghi A. Khataee A. Rezanejad F. Gohari G. (2019). Evaluating the Toxic Impacts of Cadmium Selenide Nanoparticles on the Aquatic Plant Lemna Minor. Molecules 24, 410. 10.3390/molecules24030410 PMC6385043 30678088

[B93] Tiwari A. Verma A. Panda P. K. Saraf S. Jain A. Jain S. K. (2019). “Stimuli-responsive Polysaccharides for colon-targeted Drug Delivery,” in Stimuli Responsive Polymeric Nanocarriers for Drug Delivery Applications (Elsevier), 547–566. 10.1016/b978-0-08-101995-5.00022-2

[B94] Turner M. R. Balu-Iyer S. V. (2018). Challenges and Opportunities for the Subcutaneous Delivery of Therapeutic Proteins. J. Pharm. Sci. 107, 1247–1260. 10.1016/j.xphs.2018.01.007 29336981 PMC5915922

[B95] Tzankov B. Yoncheva K. Popova M. Szegedi A. Momekov G. Mihály J. (2013). Indometacin Loading and *In Vitro* Release Properties from Novel Carbopol Coated Spherical Mesoporous Silica Nanoparticles. Microporous mesoporous Mater. 171, 131–138. 10.1016/j.micromeso.2012.12.037

[B96] Vadia N. Rajput S. (2011). Mesoporous Material, MCM-41: a New Drug Carrier. Asian J. Pharm. Clin. Res. 4, 44–53.

[B97] Vallet‐Regí M. Balas F. Arcos D. (2007). Mesoporous Materials for Drug Delivery. Angew. Chem. Int. Edition 46, 7548–7558. 10.1002/anie.200604488 17854012

[B98] Vallet-Regí M. Colilla M. Izquierdo-Barba I. Manzano M. (2018). Mesoporous Silica Nanoparticles for Drug Delivery: Current Insights. Molecules 23, 47. 10.3390/molecules23010047 PMC5943960 29295564

[B99] Vallet-Regí M. (2012). Mesoporous Silica Nanoparticles: Their Projection in Nanomedicine. Int. Scholarly Res. Notices, 2012. 10.5402/2012/608548

[B100] Voiriot G. Philippot Q. Elabbadi A. Elbim C. Chalumeau M. Fartoukh M. (2019). Risks Related to the Use of Non-steroidal Anti-inflammatory Drugs in Community-Acquired Pneumonia in Adult and Pediatric Patients. Jcm 8, 786. 10.3390/jcm8060786 PMC6617416 31163625

[B101] Wang L.-S. Wu L.-C. Lu S.-Y. Chang L.-L. Teng I.-T. Yang C.-M. (2010). Biofunctionalized Phospholipid-Capped Mesoporous Silica Nanoshuttles for Targeted Drug Delivery: Improved Water Suspensibility and Decreased Nonspecific Protein Binding. ACS nano 4, 4371–4379. 10.1021/nn901376h 20731423

[B102] Wang S. (2009). Ordered Mesoporous Materials for Drug Delivery. Microporous mesoporous Mater. 117, 1–9. 10.1016/j.micromeso.2008.07.002

[B103] Wang X. Zhang Y. Luo W. Elzatahry A. A. Cheng X. Alghamdi A. (2016). Synthesis of Ordered Mesoporous Silica with Tunable Morphologies and Pore Sizes *via* a Nonpolar Solvent-Assisted Stöber Method. Chem. Mater. 28, 2356–2362. 10.1021/acs.chemmater.6b00499

[B104] Wang Y.-P. Zhou P. Luo S.-Z. Liao X.-P. Wang B. Shao Q. (2018). Controllable Synthesis of Monolayer Poly(acrylic Acid) on the Channel Surface of Mesoporous Alumina for Pb(II) Adsorption. Langmuir 34, 7859–7868. 10.1021/acs.langmuir.8b00789 29863877

[B105] Wu S.-H. Mou C.-Y. Lin H.-P. (2013). Synthesis of Mesoporous Silica Nanoparticles. Chem. Soc. Rev. 42, 3862–3875. 10.1039/c3cs35405a 23403864

[B106] Xiao Song X. Hu L. Pang X. Li S. (2019). Synthesis of a Novel Mesoporous Carbon Nanocube@Mesoporous Silica@Poly(acrylic Acid) Composite and Application as Potential Drug Carriers. Russ. J. Phys. Chem. 93, 1349–1356. 10.1134/s003602441907029x

[B107] Xu C. Lei C. Yu C. (2019). Mesoporous Silica Nanoparticles for Protein protection and Delivery. Front. Chem. 7, 290. 10.3389/fchem.2019.00290 31119124 PMC6504683

[B108] Yamada S. Shang Y. Yamada I. Tagaya M. (2019). Synthesis of Phosphonate-Containing Mesoporous Silica Spheres under Basic Condition. Adv. Powder Tech. 30, 1116–1119. 10.1016/j.apt.2019.02.021

[B109] Yeganeh M. Asadi N. Omidi M. Mahdavian M. (2019). An Investigation on the Corrosion Behavior of the Epoxy Coating Embedded with Mesoporous Silica Nanocontainer Loaded by Sulfamethazine Inhibitor. Prog. Org. Coat. 128, 75–81. 10.1016/j.porgcoat.2018.12.022

[B110] Yi H. (2021). Secure Social Internet of Things Based on post-quantum Blockchain. Piscataway, NJ, United States: IEEE transactions on Network Science and Engineering.

[B111] Yin L. Zhang X. (2020). Green and Quality Development of Service Industry in West Coast Economic Zone. J. Coastal Res. 103, 1158–1161. 10.2112/si103-242.1

[B112] Yin W. Liu L. Zhang H. Tang S. Chi R. (2020). A Facile Solvent-free and One-step Route to Prepare Amino-Phosphonic Acid Functionalized Hollow Mesoporous Silica Nanospheres for Efficient Gd(III) Removal. J. Clean. Prod. 243, 118688. 10.1016/j.jclepro.2019.118688

[B113] Yu J. Cao J. Du L. Wei Y. Wang T. Tian H. (2018). Enhancement of Diethyl Malonate Hydrogenation to 1,3-propanediol Using Mesoporous Cu/SBA-15 as Catalyst. Appl. Catal. A: Gen. 555, 161–170. 10.1016/j.apcata.2018.02.020

[B114] Yue Y.-H. Gédéon A. Bonardet J.-L. d'Espinose J. B. Melosh N. Fraissard J. (2000). “Direct Incorporation of A1 in SBA Mesoporous Materials: Characterization, Stability and Catalytic Activity,” in Studies in Surface Science and Catalysis (Elsevier), 209–218. 10.1016/s0167-2991(00)80216-5

[B115] Zeleňák V. Hornebecq V. Llewellyn P. (2005). Zinc (II)-benzoato Complexes Immobilised in Mesoporous Silica Host. Microporous mesoporous Mater. 83, 125–135.

[B116] Zeng W. Qian X.-F. Zhang Y.-B. Yin J. Zhu Z.-K. (2005). Organic Modified Mesoporous MCM-41 through Solvothermal Process as Drug Delivery System. Mater. Res. Bull. 40, 766–772. 10.1016/j.materresbull.2005.02.011

[B117] Zhang W. Zheng N. Chen L. Xie L. Cui M. Li S. (2019). Effect of Shape on Mesoporous Silica Nanoparticles for Oral Delivery of Indomethacin. Pharmaceutics 11, 4. 10.3390/pharmaceutics11010004 PMC6359657 30583601

[B118] Zhang W.-H. Lu X.-B. Xiu J.-H. Hua Z.-L. Zhang L.-X. Robertson M. (2004). Synthesis and Characterization of Bifunctionalized Ordered Mesoporous Materials. Adv. Funct. Mater. 14, 544–552. 10.1002/adfm.200305001

[B119] Zhao D. Sun J. Li Q. Stucky G. D. (2000). Morphological Control of Highly Ordered Mesoporous Silica SBA-15. Chem. Mater. 12, 275–279. 10.1021/cm9911363

[B120] Zhao P. Jiang H. Jiang T. Zhi Z. Wu C. Sun C. (2012). Inclusion of Celecoxib into Fibrous Ordered Mesoporous Carbon for Enhanced Oral Bioavailability and Reduced Gastric Irritancy. Eur. J. Pharm. Sci. 45, 639–647. 10.1016/j.ejps.2012.01.003 22251657

[B121] Zhou S. Wu D. Yin X. Jin X. Zhang X. Zheng S. (2017). Intracellular pH-Responsive and Rituximab-Conjugated Mesoporous Silica Nanoparticles for Targeted Drug Delivery to Lymphoma B Cells. J. Exp. Clin. Cancer Res. 36, 24–14. 10.1186/s13046-017-0492-6 28166836 PMC5292796

[B122] Zhu S. Zhang D. Yang N. (2009). Hydrophobic Polymers Modification of Mesoporous Silica with Large Pore Size for Drug Release. J. Nanopart Res. 11, 561–568. 10.1007/s11051-007-9325-4

[B123] Zhu Y. Shi J. Chen H. Shen W. Dong X. (2005). A Facile Method to Synthesize Novel Hollow Mesoporous Silica Spheres and Advanced Storage Property. Microporous Mesoporous Mater. 84, 218–222. 10.1016/j.micromeso.2005.05.001

